# Supplementary material for: Content-rich biological network constructed by mining PubMed abstracts
Source: BMC Bioinformatics. 2004 Oct 8;5:147. doi: 10.1186/1471-2105-5-147 (PMC528731; doi:10.1186/1471-2105-5-147)
Supplement: Additional File 2 — The original results of the above study (non-essential files are deleted to keep the file size under the limit set by BMC bioinformatics). [file 1471-2105-5-147-S2.bz2 › chilibotAdditionalFile2/dip05/306ID9117348E1304/html/CD44_CD9.html]

 


 **CD44** and **CD9** 
  
Found 109 abstracts in PubMed, retrieved 05.  
 

 What does Google say? 
 PDF only 
| .edu only 

---

**Interactive relationship** (e.g. stimulation, inhibition, etc)

**Non-interactive relationship** (e.g. studied together, co-existance, homology, etc.)

- Based on their immunophenotype, the cells are relatively homogeneous, with shared expression of integrin beta 1, the hyaluronate receptor  [ **CD44** ] , and the tetraspan protein  **CD9** , among other markers.  Ref: 12880371 Expert Opin Biol Ther, 2003
- The expression, of multidrug resistance MDR associated proteins PGP  [ **CD44** ] , LRP, MRP1  [ **CD9** ]  was also analyzed.  Ref: 12801292 Eur J Haematol, 2003
- To address whether multidrug resistance protein MRP affects GO susceptibility we characterized Pgp  [ **CD44** ] , MRP1  [ **CD9** ] , and MRP2 expression in CD33 cell lines and CD33 AML samples and analyzed the effect of the Pgp  [ **CD44** ]  inhibitor cyclosporine CSA and the MRP inhibitor MK 571 on GO induced cytotoxicity.  Ref: 12689934 Blood< MedlineTA>Blood, 2003
- PGP  [ **CD44** ] , LRP, and MRP1  [ **CD9** ]  expression at onset and at first relapse was low.  Ref: 12801292 Eur J Haematol, 2003
- These include MDR1 P glycoprotein, PgP  [ **CD44** ] , the multidrug resistance related proteins MRP1  [ **CD9** ]  5, and lung resistance protein LRP.  Ref: 12699350 Curr Drug Targets, 2003
- Data obtained are presented in concurrence with Clusters of Thymic Epithelial Staining CTES classification for various monoclonal antibodies recognizing CD antigens CD1, CD1a, CD6,  **CD9** , CD14, CD16, CD29, CD30, CD32,  **CD44** , CD45RB, CD47, CD48, CD49a, CD49b, CD49c, CD49d, CD49e, CD49f, CD51, CD53, CD54, CD56, CD57, CD63, CD85, CD95, CD98, CD102, CD103, CD106, CD109, CD146, CD147, CD148, CD151, CD152, CD158a, CD158b, CD164, CD165, CD166 and for monoclonal antibodies 1B1 G7, A4, BD46, BLTZ, HP1C5, IND.64, M72, WU947 whose specifities are not yet defined.  Ref: 12725440 Ann Anat, 2003
